# Fluorescence in situ hybridization karyotyping reveals the presence of two distinct genomes in the taxon *Aegilops tauschii*

**DOI:** 10.1186/s12864-017-4384-0

**Published:** 2018-01-02

**Authors:** Laibin Zhao, Shunzong Ning, Yingjin Yi, Lianquan Zhang, Zhongwei Yuan, Jirui Wang, Youliang Zheng, Ming Hao, Dengcai Liu

**Affiliations:** 1 Triticeae Research Institute, Sichuan Agricultural University, No. 211 Huiming Rd, Wenjiang District, Chengdu City, Sichuan province 611130 People’s Republic of China; 2 Key Laboratory of Crop Genetic Resources and Improvement, Ministry of Education, Sichuan Agricultural University, Yaan, Sichuan 625014 China

**Keywords:** Chromosome differentiation, D genome, Repeat sequences, Spike morphology, Subspecies, Wheat evolution

## Abstract

**Background:**

*Aegilops tauschii* is the donor of the bread wheat D genome. Based on spike morphology, the taxon has conventionally been subdivided into ssp. *tauschii* and ssp. *strangulata*. The present study was intended to address the poor match between this whole plant morphology-based subdivision and genetic relationships inferred from genotyping by fluorescence in situ hybridization karyotyping a set of 31 *Ae. tauschii* accessions.

**Results:**

The distribution of sites hybridizing to the two probes oligo-pTa-535 and (CTT)_10_ split the *Ae. tauschii* accessions into two clades, designated D^t^ and D^s^, which corresponded perfectly with a previously assembled phylogeny based on marker genotype. The D^t^ cluster was populated exclusively by ssp. *tauschii* accessions, while the D^s^ cluster harbored both ssp. *strangulata* and morphologically intermediate accessions. As a result, it is proposed that *Ae. tauschii* ssp. *tauschii* is restricted to carriers of the D^t^ karyotype: their spikelets are regularly spaced along the rachis, at least in the central portion of their spike. Accessions classified as *Ae. tauschii* ssp. *strangulata* carry the D^s^ karyotype; their spikelets are irregularly spaced. Based on this criterion, forms formerly classified as ssp. *tauschii* var. *meyeri* have been re-designated ssp. *strangulata* var. *meyeri*.

**Conclusions:**

According to the reworking of the taxon, the bread wheat D genome was most probably donated by ssp. *strangulata* var. *meyeri*. Chromosomal differentiation reveals intra-species taxon of *Ae. tauschii*. *Ae. tauschii* ssp. *tauschii* has more distant relationship with breed wheat than ssp. *strangulata* and can be used for breeding improving effectively.

## Background

The range of diploid goatgrass (*Aegilops tauschii*) stretches from Turkey in the west to China in the east. The species is adapted to a wide diversity of environments [1]. Most significantly, it is understood to be the donor of the bread wheat D genome [2, 3], as well as being a pivotal genome in several *Aegilops* tetraploid and hexaploid species [4]. Based on variation in spike morphology, the species has conventionally been subdivided into ssp. *tauschii* and ssp*. strangulata*; members of the former subspecies develop elongated cylindrical spikelets, while those of the latter form moniliform spikes bearing quadrate spikelets [5–8]. Within ssp. *tauschii* two distinct varieties have been recognized, namely var. *anathera* and var. *meyeri*. The diagnostic character for the former variety is awnlessness, while the latter types form slender, short spikes and develop only 4-8 spikelets per spike [5, 8]. This classification remains controversial because of the existence of morphologically intermediate types [1, 9–11]. For example, individuals forming mildly moniliform spikes have been classified as ssp. *strangulata,* even though this taxon is restricted to individuals which form a sharply defined moniliform spike [10, 12].

The established subspecies structure is not well matched with genetic relationships derived from genotypic characterization. Two distinct phylogenetic lineages, designated L1 and L2, have been recognized [13–15]. The former coincides with ssp. *tauschii* [13], but the latter includes, along with ssp. *strangulata*, accessions formerly assigned to ssp. *tauschii* [13, 15, 16]. The most troublesome taxon is var. *meyeri*, which has been assigned to ssp. *tauschii* on the basis of spike morphology [11] but appears to be genetically more closely related to ssp. *strangulata* [13, 16–18].

Much of the large (4 Gbp) *Ae. tauschii* genome consists of repetitive DNA [19–22]. When C-banding was exploited for karyotypic analysis, the conclusion was that the bread wheat D genome was more similar to the one present in ssp. *strangulata* than the one in ssp. *tauschii* [23, 24]. A more sophisticated karyotyping tool is represented by fluorescence in situ hybridization (FISH), and this approach has been applied with great success to identify individual wheat chromosomes, based on the distribution of sites of hybridization with probes designed to detect a number of repetitive sequences [25–31]. Here, the purpose was to use FISH to enable an objective, karyotype-based analysis of the subspecies structure of *Ae. tauschii.*

## Methods

### Plant materials

**Fig. 1:**
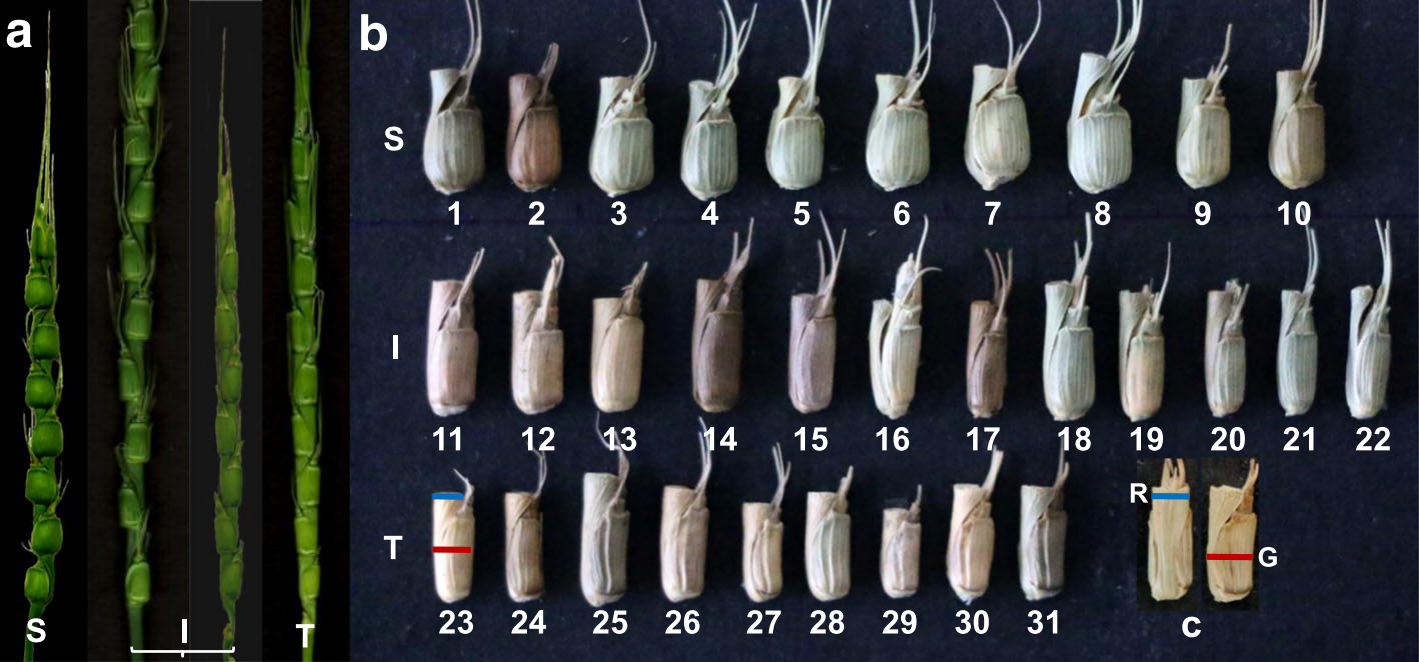
Morphological variation within *Ae. tauschii.* (a) Spikes, (b, c) spikelets. Types S (ssp. *strangulata*), T (ssp. *tauschii*) and I (intermediate forms) are distinguished by the width of the rachis segment (blue bar in #23) and the glume (red bar in #23). In (c) the measurement points for spikelet glume width (G) and rachis segment width (R) used to derive SI are indicated. The numbers 1-31 in (b) relate to accessions 1-31 in Table 1

The bread wheat cultivar Chinese Spring (CS) and a collection of 31 *Ae. tauschii* accessions (Table 1) were subjected to FISH karyotyping. The spike morphology of the *Ae. tauschii* accessions was observed more than ten years at Wenjiang, Dujiangyan and Yaan experimental station of Sichuan Agricultural University. Given the existence of mildly moniliform spike types, an objective criterion, termed the subspecies index (SI), was also used to score spike morphology: the SI represents the ratio between the widths of the glume and the rachis segment measured on a spikelet sampled from the central portion of the spike. Individuals classified as ssp. *tauschii* have a low SI, while those belonging to ssp. *strangulata* have a high SI [32, 33]. Here, the measurement points for spikelet glume width (G) and rachis segment width (R) used to derive SI are indicated in Fig. 1. We measured the glume width in side face rather than in front face [32]. The mean SI obtained from about ten spikelets was used. Because the spikes morphology of each accession from the three different geographical fields were no obvious variation, only the spikes collected from Wenjiang experimental station were used to analyze. Least significant difference (LSD) was used for mean comparison at the probability of *P* < 0.01 by SPSS V20 (International Business Machines Corp., NY, USA).

**Table 1:** *Aegilops tauschii* accessions

Code	Accession^a^	Origin	Collector’s taxon	Spike type (SI ± SD)^b^	FISH group	Our taxon	Sublineage^c^
1	AS2388	Iran	ssp. *strangulata*	S (1.72 ± 0.10)^ABCD^	D^s^	ssp. *strangulata*	L2E
2	PI603227	Iran	ssp. *strangulata*	S (1.59 ± 0.07)^EF^	D^s^	ssp. *strangulata*	L2E
3	CIae13	Iran	ssp. *strangulata*	S (1.69 ± 0.09)^BCDE^	D^s^	ssp. *strangulata*	L2E
4	CIae16	Iran	ssp. *strangulata*	S (1.73 ± 0.11)^ABC^	D^s^	ssp. *strangulata*	L2E
5	CIae18	Iran	ssp. *strangulata*	S (1.74 ± 0.10)^ABC^	D^s^	ssp. *strangulata*	L2E
6	AS2386	Iran	ssp. *strangulata*	S (1.85 ± 0.14)^A^	D^s^	ssp. *strangulata*	–
7	AS2403	unknown	ssp. *strangulata*	S (1.79 ± 0.08)^AB^	D^s^	ssp. *strangulata*	L2E
8	AS2405	Iran	ssp. *strangulata*	S (1.77 ± 0.04)^ABC^	D^s^	ssp. *strangulata*	L2E
9	AS2402	Israel	ssp. *strangulata*	S (1.66 ± 0.07)^CDE^	D^s^	ssp. *strangulata*	L2E
10	AS66	Transcaucasia	ssp. *strangulata*	S (1.76 ± 0.09)^ABC^	D^s^	ssp. *strangulata*	L2E
11	PI603238	Azerbaijan	ssp. *strangulata*	I (1.40 ± 0.04)^G^	D^s^	ssp. *strangulata*	L2 W
12	PI574465	Azerbaijan	ssp. *strangulata*	I (1.46 ± 0.09)^G^	D^s^	ssp. *strangulata*	L2 W
13	PI431602	Turkmenistan	ssp. *strangulata*	I (1.41 ± 0.07)^FG^	D^s^	ssp. *strangulata*	L2 W
14	PI603249	Iran	ssp. *strangulata*	I (1.60 ± 0.05)^EF^	D^s^	ssp. *strangulata*	L2 W
15	AL8/78	Armenia	ssp. *strangulata*	I (1.41 ± 0.05)^G^	D^s^	ssp. *strangulata*	L2 W
16	PI603239	Azerbaijan	ssp. *tauschii*	I (1.67 ± 0.12)^BCDE^	D^s^	ssp. *strangulata*	L2 W
17	PI603233	Azerbaijan	ssp. *tauschii*	I (1.41 ± 0.07)^G^	D^s^	ssp. *strangulata*	L2 W
18	PI276985	Iran	ssp. *tauschii* var. *meyeri*	I (1.74 ± 0.20)^ABC^	D^s^	ssp. *strangulata* var. *meyeri*	L2E
19	CIae26	Iran	ssp. *tauschii* var. *typica*	I (1.46 ± 0.05)^G^	D^s^	ssp. *strangulata*	L2E
20	AS63	unknown	ssp. *tauschii* var. *meyeri*	I (1.49 ± 0.07)^FG^	D^s^	ssp. *strangulata* var. *meyeri*	L2E
21	CIae23	Iran	ssp. *tauschii* var. *meyeri*	I (1.59 ± 0.04)^EF^	D^s^	ssp. *strangulata* var. *meyeri*	L2E
22	CIae21	Iran	ssp. *tauschii* var. *typica*	I (1.70 ± 0.07)^BCDE^	D^s^	ssp. *strangulata*	L2E
23	PI210987	Afghanistan	ssp. *tauschii*	T (1.18 ± 0.03)^H^	D^t^	ssp. *tauschii*	L1E
24	PI574467	Russian Federation	ssp. *tauschii*	T (1.04 ± 0.04)^I^	D^t^	ssp. *tauschii*	L1 W
25	AS79	China	ssp. *tauschii*	T (1.21 ± 0.03)^H^	D^t^	ssp. *tauschii*	L1E
26	AS77	China	ssp. *tauschii*	T (1.18 ± 0.03)^H^	D^t^	ssp. *tauschii*	–
27	AS2410	China	ssp. *tauschii*	T (1.19 ± 0.04)^H^	D^t^	ssp. *tauschii*	–
28	AS60	Iran	ssp. *tauschii*	T (1.22 ± 0.05)^H^	D^t^	ssp. *tauschii*	L1E
29	AS67	Iran	ssp. *tauschii*	T (1.27 ± 0.04)^H^	D^t^	ssp. *tauschii*	L1E
30	AS68	Iran	ssp. *tauschii*	T (1.25 ± 0.09)^H^	D^t^	ssp. *tauschii*	L1E
31	CIae1	Pakistan	ssp. *tauschii*	T (1.17 ± 0.04)^H^	D^t^	ssp. *tauschii*	L1E

^a^: Accessions marked “AS” were obtained from the Triticeae Research Institute, Sichuan Agricultural University, China; those marked “PI” or “CIae” were obtained from USDA-ARS. ^b^: Types S (ssp. *strangulata*), T (ssp. *tauschii*) and I (intermediate forms). The SI was given by the ratio spikelet glume width/rachis segment width. Capital letters after the average SI value and standard deviations (SD) denote the results of LSD comparison (*P* < 0.01). ^c^: Genetic lineage data according to marker genotype taken from Wang et al. (2013). L1E (L1 East) and L1 W (L1 West) represent the T type gene pool, and L2E (L2 East) and L2 W (L2 West) the S type gene pool

### FISH karyotyping

Caryopses were imbibed for 24 h at 4 °C, then germinated under a 16 h photoperiod (light/dark temperature 22/16 °C). Root tips of length 1-2 cm were excised and exposed for 2 h to 1.0 MPa NO gas, fixed in glacial acetic acid for at least 5 min, and finally stored in 70% *v*/v ethanol before preparing slides. The slides were prepared as previously described by Komuro et al. [30] and then denaturated as described by Hao et al. [34]. A 10 μL aliquot of hybridization mixture was applied to each slide, which was allowed to incubate at 37 °C for least one hour. Finally, a drop of DAPI-containing Vectashield mounting medium (Vector Laboratories, Inc., Burlingame, CA, USA) was added and the preparation was covered with a coverslip. Hybridization signals were observed using an Olympus BX-51 epifluorescence microscope and the images were taken using a Photometric SenSys Olympus DP70 CCD camera (Olympus, Tokyo). Raw images were processed using Photoshop V7.0 (Adobe Systems Incorporated, San Jose, CA). Once the slide had been scored, it was readied for a subsequent stripping and rehybridization following Komuro et al. [30]. The probes included in the hybridization solution were 6-carboxyfluorescein (6-FAM) or 6-carboxytetramethylrhodamine (Tamra) labeled oligonucleotides (CTT)_10_, (AAC)_5_ and (ACG)_5_ [28, 35, 36], oligo-pSc119.2, oligo-pTa-535, oligo-pTa71 [31] and oligo-pTa-713 [37], all synthesized by TSINGKE Biological Technology Company (Chengdu, Sichuan, China).

## Results

### The spike morphology of *Ae. tauschii*

Based on the appearance of the spike, the 31 *Ae. tauschii* accessions were classified into three morphological types (Table 1; Fig. 1) as follows:

Type S (ten accessions): these accessions formed markedly moniliform spikes with quadrate spikelets (similar with respect to width and length). Their average SI values ranged from 1.66-1.85 in 2015 in Wenjiang. Individual spikelets of these accessions ranged from 1.52-2.13. The members of this group all belong to ssp. *strangulata.*

Type T (nine accessions): these accessions formed elongated cylindrical spikes. Their low SI (1.04-1.27) reflected the similar width of their spikelet glume and rachis segment. Individual spikelets of these accessions ranged from 1.00-1.36. The members of this group all belong to ssp. *tauschii.*

Type I (12 accessions): these accessions were classified as intermediate types, with an SI ranging from 1.40-1.74. Individual spikelets of these accessions ranged from 1.33-2.09. Some (PI603238, PI574465, PI431602, PI603249 and AL8/78) had previously been assigned to ssp. *strangulata* on the basis of their mildly moniliform spike, but their spikelets were too elongated to be unequivocally assigned to this taxon. Accessions PI276985, AS63 and CIae23 (assigned previously to ssp. *tauschii* var. *meyeri)* formed elongated spikelets, but their spikes were not sufficiently cylindrical and their rachis segments too narrow to fit this taxon.

### FISH markers specific for two typical ssp.

**Fig. 2:**
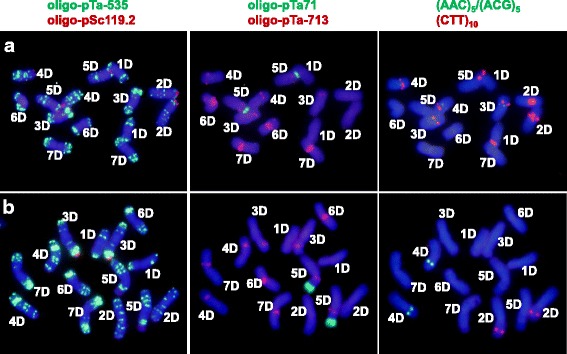
FISH karyotype, based on seven probes, of (**a**) the Type S accession AS2402, (**b**) the Type T accession AS79

Seven probes (Fig. 2) were preliminarily hybridized in situ to mitotic chromosome spreads of nine accessions, including the two from S type (AS2388 and AS2402), two from I type (AS63 and PI431602), and five from T type (AS68, AS77, AS79, CIae1 and PI210987). However, the probes oligo-pTa-713, (AAC)_5_ and (ACG)_5_ failed to reveal any subspecies-specific difference for the analyzed materials. As a result, these three probes were not used any further. Although oligo-pSc119.2 also failed to detect subspecies-specific difference, it was helpful to identify chromosomes 2D, 3D, and 4D. Oligo-pSc119.2 and the remaining three probes were tested on mitotic chromosome spreads of the full set of *Ae. tauschii* accessions as well as on CS.

#### Oligo-pTa71

**Fig. 3:**
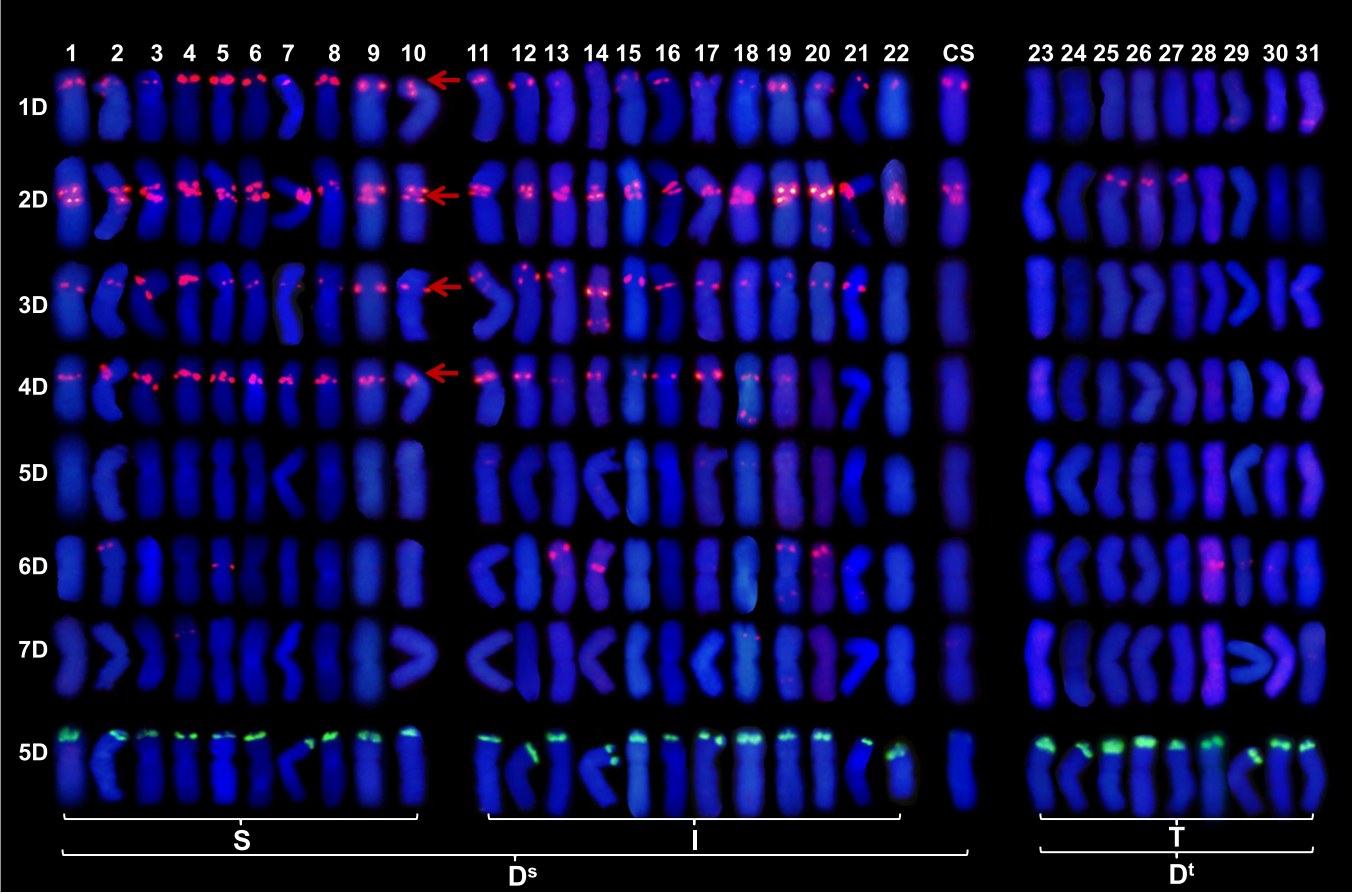
FISH karyotyping of 31 accessions of *Ae. tauschii* and the D genome of CS, derived from hybridization with (CTT)_10_ (red) and oligo-pTa71 (green). Type S: ssp. *strangulata*, Type T: ssp. *tauschii,* Type I: intermediate forms. Sites specific for Type S chromosomes are shown by red arrows. The numbers 1-31 in (b) relate to accessions 1-31 in Table 1

This sequence hybridized to the nucleolar organizing region on chromosome arm 5DS in all of the *Ae. tauschii* accessions. The hybridization signal strength was higher in the Type T than in the Type S accessions (Fig. 3). However, the probe did not hybridize strongly with the CS 5D chromosome.

#### Oligo-pSc119.2

**Fig. 4:**
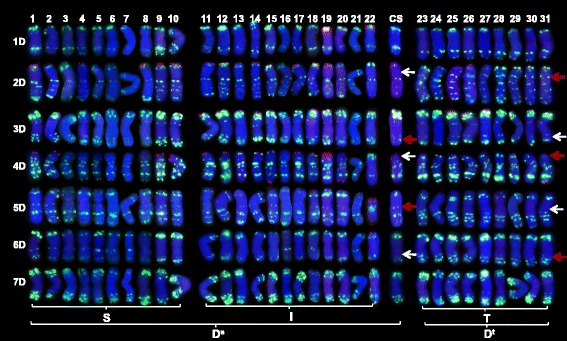
FISH karyotyping of 31 accessions of *Ae. tauschii* and the D genome of CS, derived from hybridization with oligo-pSc119.2 (red) and oligo-pTa-535 (green). Type S: ssp. *strangulata*, Type T: ssp. *tauschii,* Type I: intermediate forms. The white arrows indicate sites which are absent, and the red arrows sites which are present for FISH signals. The numbers 1-31 in (b) relate to accessions 1-31 in Table 1

This probe hybridized to sites close to the telomere of chromosome arms 1DS, 2DS, 3DS and 4DS (Fig. 4). The pattern of hybridization did not discriminate between the Types S, T and I, but was useful as a means of identifying individual chromosomes.

#### (CTT)_10_

This probe hybridized to sites on chromosome arms 1DS, 2DS, 3DS and 4DS, and the patterns were informative with respect to the three Types (Fig. 3). The four chromosome arms harbored a hybridization site in all Type S accessions, but these sites were absent from all Type T accession karyotypes*.* A site in the sub-telomeric region of chromosome arm 1DL was present in both the Type T accessions AS67 and AS68 (from Iran) and CIae1 (Pakistan). A further site in the sub-telomeric region of chromosome arm 2DS was restricted to just three Type T accessions (AS77, AS79, AS2410) all originating from central China, the most easterly reach of the species.

#### Oligo-pTa-535

The oligo-pTa-535 probe hybridized to many chromosomal sites, allowing it to be used as a means discriminating each of the seven of *Ae. tauschii* chromosomes (Fig. 4). Type-specific sites were observed on chromosome arms 2DS, 3DL, 4DS, 5DL and 6DL. Both the middle region of chromosome arm 2DS and the sub-telomeric region of chromosome arm 4DS included hybridization sites which were unique to Type T accessions, while one site in the sub-telomeric region of chromosome arm 3DL and another in the centromeric region of 5DL were absent from all Type T accessions. The 5DL arm harbored several hybridization sites, but their distribution along the arm differed between Type S and Type T accessions. A similar Type-specific distribution of sites was detected in the region stretching from about two thirds of the way along chromosome arm 6DL to its telomere.

### The FISH karyotype of type S and type I accessions

**Fig. 5:**
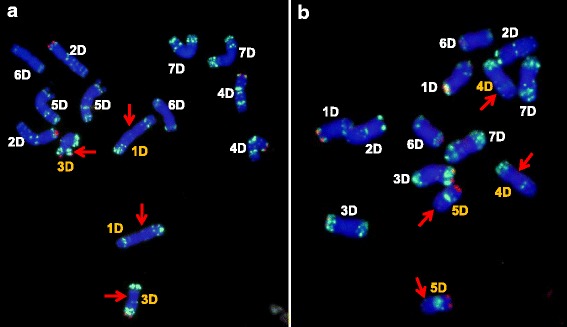
Chromosome variants with oligo-pSc119.2 (red) and oligo-pTa-535 (green). **a** #14 (PI603249) harbors a longer chromosome 1D and a shorter chromosome 3D; **b** #22 (CIae 21) harbors a shorter chromosome 5D, and a version of chromosome 4D showing an unusual distribution of hybridization sites

The karyotypes of Type S and Type I accessions were quite similar to one another. The four (CTT)_10_ sites present in all Type S (but in no Type T) accessions were represented in most of the Type I karyotypes; the exceptions were the chromosome arm 1DS site (missing in PI603249), the 3DS site (missing in PI603249 and CIae21) and the 4DS site (missing in CIae21, AS63 and CIae23) (Fig. 3). The absence of the 1DS and 3DS sites in PI603249 was accompanied by lengthened chromosome 1D, a foreshortened chromosome 3D and a unique hybridization site on chromosome 3D (Fig. 5a), which might be indicative of a major structural alteration in the genome. In CIae21, both chromosomes 4D and 5D were unusual in length (Fig. 5b). All three Type I accessions lacking the 4DS site formed slender spikelets (Fig. 1), used as a diagnostic for var. *meyeri*, although CIae21 was classified as var. *typica* by its collector (Table 1). The sub-telomeric region of chromosome arm 6DS of the three Type I accessions PI431602, CIae26 and AS63, along with that of the Type S accession PI603227, harbored a further (CTT)_10_ site (Fig. 3). The distribution of oligo-pTa-535 sites confirmed the closeness of the relationship between Types S and I*.* In both the middle region of chromosome arm 2DS and the sub-telomeric region of chromosome arm 4DS, sites present in the Type T karyotype were missing in both Type S and I, while the Type S-specific sites close to the chromosome arm 3DL telomere were also present in Type I (Fig. 4). The weakly hybridizing sites lying on chromosome arm 5DL close to the centromere were present in both Type S and I accessions*.* Similarly, the chromosome arm 6DL region was more similar between Types S and I than between Types S and T. Overall, the karyotypic analysis provided evidence that Type I accessions are genetically closer to Type S than to Type T.

### The FISH karyotype of *Ae. tauschii* in relation to that of CS

Probe (CTT)_10_ hybridized to sites on chromosome arms 1DS and 2DS in the Type S and I accessions and also to the same location in CS (Fig. 3). However, neither the chromosome arm 3DS nor 4DS sites were present in CS*,* which was also a feature of the Type I accession CIae21, while AS63 and CIae23 lacked the 4DS site, but harbored the 3DS one. The (CTT)_10_ sub-telomeric sites on 7DS were only present in the Type S accession CIae16, Type I accession PI276985, and CS (Fig. 3). With respect to oligo-pTa-535, the karyotype of CS (as also that of the Type S and Type I accessions) lacked the sites at middle region on 2DS and the sub-telomeric region of 4DS, and shared a similar distribution of sites with the Types S and I accessions on chromosome arms 3DL, 5DL and 6DL and around the chromosome 5D centromere (Fig. 4).

## Discussion

Although it was thought at one time that repetitive DNA had no function, it is now believed that it does play some role in chromosome organization and stabilization, mitosis and meiosis, and even gene regulation [38]. Both the amount and distribution of such sequences act to drive evolution and speciation [39], so that their diversification has been used to assess genetic relatedness at both the species and the genome level [40, 41].

### Genome heterogeneity within *Ae. tauschii*

FISH karyotyping based on two sequences (oligo-pTa-535 and (CTT)_10_) was able to reveal intraspecific differentiation within *Ae. tauschii.* Two distinct forms of the D genome were recognized from the karyotypic analysis of the 31 accessions (Figs. 3 and 4). One, present in nine accessions categorized as ssp. *tauschii*, is designated here D^t^, and the other, present in the ssp. *strangulata* and morphologically intermediate forms, as D^s^. Previous analyses based on marker genotype have also proposed the presence of two lineages [13–15]. Of the 31 accessions represented in the test germplasm set, 28 were also genotyped by Wang et al. 2013; a comparison of the outcomes shows the D^t^ and D^s^ designations to be fully congruent with, respectively, lineages L1 and L2 (Table 1). L1 members (seven accessions) all form spikes of the ssp. *tauschii* type (Type T). Meanwhile L2, according to the collectors’ classification, consists of seven accessions designated as ssp. *tauschii* and 14 as ssp. *strangulata* [15]; a more exacting observation, however (see Fig. 1), has suggested that all of these seven supposed ssp. *tauschii* accessions, along with five of the 14 supposed ssp. *strangulata* accessions exhibit a spike morphology which better fits the intermediate type (Type I). The conclusion is that L1/D^t^ corresponds to ssp. *tauschii* and L2/D^s^ to either ssp. *strangulata* or the intermediate type (Table 1).

### A re-consideration of intra-species taxa within *Ae. tauschii*

*Ae. tauschii* has conventionally been sub-divided into the two subspecies *tauschii* and *strangulata* [5, 8], which leaves unclear the status of the morphologically intermediate forms. The present proposal is that membership of ssp. *tauschii* should be based on the formation of elongated cylindrical spikes and a low SI (Fig. 1), while the rest of the taxon, which includes both ssp. *strangulata* sensu stricto and intermediate forms, are classified as ssp. *strangulata*. Accordingly, ssp. *tauschii* var. *meyeri*, as an intermediate form, should be re-designated ssp. *strangulata* var. *meyeri*; this would fit with the genotypic data, which suggests that var. *meyeri* individuals are genetically closely related to ssp. *strangulata* [13, 15–18]. The suggested classification would result in a match between the botanically- and genetics-based categorizations, i.e. that ssp. *tauschii* becomes synonymous with lineage L1 (D^t^), and ssp. *strangulata* with L2 (D^s^).

### The diversification of the D genome

Of interest is the question as to how the D genome evolved the two distinct forms D^t^ and D^s^ *.* Lineages L1 and L2 are recognizably different from one another, and virtually no intermediate forms are known [15]. On the basis of an analysis of non-coding chloroplast DNA sequences, it has been suggested that neither of the two subspecies could represent a progenitor of the other [42]. Here, the proposal is that the two subspecies originated independently, following a hybridization with a third species. According to Marcussen et al. [43], the bread wheat A and B genomes diverged from a common ancestor, and later combined to give rise to the D genome via homoploid hybrid speciation. If this evolutionary model is correct, it is possible that the two forms D^t^ and D^s^ evolved either from independent hybridization events or via segregation from a common progenitor hybrid.

An analysis based on single nucleotide polymorphisms has suggested that the donor of the bread wheat D genome was a member of the L2 lineage [15], to which the nine of the ten Type S accessions along belong (Table 1). The other five L2 members (PI276985, CIae26, AS63, CIae21 and CIae23) are all Type I, now re-designated as ssp. *strangulata* var. *meyeri.* One of these (CIae23) maps closest to the bread wheat D genome [15]. Given that the distribution of (CTT)_10_ sites across the CS D genome more closely resembled that seen in CIae21, CIae23 and AS63 (Fig. 3), the conclusion is that the progenitor of the bread wheat D genome was likely a member of ssp. *strangulata* var. *meyeri.*

### Wheat D genome genetic improvement

Further genetic improvement is needed to match wheat production to an increasing global demand. Accessing novel genetic variation from wheat’s D genome wild relatives has proved to be a highly successful strategy in recent years [44]. Probably very few *Ae*. *tauschii* individuals were involved in the original natural hybrids which were the progenitors of modern bread wheat, creating a major evolutionary bottleneck. Thus, of over 7000 D genome single nucleotide polymorphism sites, about 99% have been contributed by lineage L2 [15]. The implication is that a future priority should be to drive introgression from L1, using accessions such as AS60. The Chinese cultivar Shumai 969 released in 2013 was bred by using a synthetic hexaploid formed by crossing AS60 with *Triticum turgidum* [45]. It has rapidly become a leading cultivar in Sichuan Province, China, and demonstrates the potential of carriers of the D^t^ genome for wheat improvement.

## Conclusions

According to the reworking of the taxon, the bread wheat D genome was most probably donated by ssp. *strangulata* var. *meyeri.* Chromosomal differentiation reveals intra-species taxon of *Ae. tauschii*. *Ae. tauschii* ssp. *tauschii* has more distant relationship with breed wheat than ssp. *strangulata* and can be used for breeding improving effectively.

## Abbreviations

*Ae*: *Aegilops*
CS: Chinese Spring
FISH: Fluorescence in situ hybridization
G: Glume width
R: Rachis segment width
SI: Subspecies index
